# Chrysophanol Alleviates Metabolic Syndrome by Activating the SIRT6/AMPK Signaling Pathway in Brown Adipocytes

**DOI:** 10.1155/2020/7374086

**Published:** 2020-11-12

**Authors:** Xueying Liu, Zehong Yang, Huixuan Li, Wen Luo, Wentao Duan, Junmei Zhang, Zhangzhi Zhu, Min Liu, Saimei Li, Xiaoyi Xin, Haoxiang Wu, Shaoxiang Xian, Meijing Liu, Changhui Liu, Chuangpeng Shen

**Affiliations:** ^1^The First Clinical Medical College of Guangzhou University of Chinese Medicine, 16 Airport Road, Guangzhou 510405, China; ^2^Department of Chinese Medicine, The First People's Hospital of Kashgar Prefecture, Kashgar, Xinjiang Uygur Autonomous Region, 844000, China; ^3^Department of Endocrinology, The First Affiliated Hospital of Guangzhou University of Chinese Medicine, 16 Airport Road, Guangzhou 510405, China; ^4^Artemisinin Research Center, Guangzhou University of Chinese Medicine, 12 Airport Road, Guangzhou 510405, China; ^5^Science and Technology Innovation Center, Piwei Institute, Guangzhou University of Chinese Medicine, 12 Airport Road, Guangzhou 510405, China; ^6^Department of Chinese Medicine, The First Affiliated Hospital of Xinjiang Medical University, Urumqi, Xinjiang Uygur Autonomous Region, 830011, China; ^7^Department of Cardiovascularology, The First Affiliated Hospital of Guangzhou University of Chinese Medicine, 16 Airport Road, Guangzhou 510405, China; ^8^Beijing Advanced Innovation Center for Big Data-Based Precision Medicine, School of Medicine and Engineering, Beihang University, Beijing 100191, China; ^9^School of Pharmaceutical Sciences, Guangzhou University of Chinese Medicine, 12 Airport Road, Guangzhou 510405, China

## Abstract

Chrysophanol, a primary active ingredient of *Cassia mimosoides Linn* or *Rhei radix et rhizoma*, has various pharmacological properties, including anticancer, antidiabetic, and anti-inflammatory, as well as blood lipid regulation. However, whether chrysophanol can mitigate obesity, and its underlying mechanisms remains unclear. This study investigated whether chrysophanol effects energy metabolism in high-fat diet- (HFD-) induced obese mice and fat-specific Sirtuin 6- (SIRT6-) knockout (FKO) mice, targeting the SIRT6/AMPK signaling pathway in brown and white fat tissue. Our results showed that chrysophanol can effectively inhibit lipid accumulation in vitro and reduce mice's body weight, improve insulin sensitivity and reduced fat content of mice, and induce energy consumption in HFD-induced obese mice by activating the SIRT6/AMPK pathway. However, a treatment with OSS-128167, an SIRT6 inhibitor, or si-SIRT6, SIRT6 target specific small interfering RNA, in vitro blocked chrysophanol inhibition of lipid accumulation. Similar results were obtained when blocking the AMPK pathway. Moreover, in the HFD-induced obese model with SIRT6 FKO mice, histological analysis and genetic test results showed that chrysophanol treatment did not reduce lipid droplets and upregulated the uncoupling protein 1 (UCP1) expression. Rather, it upregulated the expression of thermogenic genes and activated white fat breakdown by inducing phosphorylation of adenosine 5′-monophosphate- (AMP-) activated protein kinase (AMPK), both in vitro and in vivo. OSS-128167 or si-SIRT6 blocked chrysophanol's upregulation of peroxisome proliferator-activated receptor-*γ* coactivator-1*α* (Pgc-1*α*) and Ucp1 expression. In conclusion, this study demonstrated that chrysophanol can activate brown fat through the SIRT6/AMPK pathway and increase energy consumption, insulin sensitivity, and heat production, thereby alleviating obesity and metabolic disorders.

## 1. Introduction

Obesity is characterized by excessive accumulation of fat, which is associated with a higher risk of insulin resistance, inflammation, type 2 diabetes, hypertension, and cardiovascular disease [1]. This multifactorial disease develops due to a long-term imbalance between regular energy intake and energy expenditure. High-fat-diet (HFD) induces obesity in mice, which is accompanied with fat deposition, oxidative stress, inflammation, and liver fibrosis. In addition, obesity, diabetes, hyperlipidemia, and hypertension can induce nonalcoholic steatohepatitis [2].

The adipose tissue is closely associated with energy metabolism. Understanding the physiological mechanism of different types of fat storage can provide theoretical basis for solving obesity. Although, in the past, the adipose tissue was known only as a fat storage organ; recently, it is also identified as a thermogenic organ, contributing to energy expenditure through the action of specialized, heat-producing brown or beige adipocytes [3, 4]. Moreover, brown adipocytes, which appear in response to thermogenic stimuli in white fat, obtained their name due to the so-called “browning” process [5]. The brown adipose tissue (BAT) and beige fat can maintain body temperature homeostasis, regulate energy balance and body weight, and influence glucose and lipid metabolism in rodents [6, 7]. The activities of BAT and beige cells negatively correlated with blood glucose concentration, insulin sensitivity, and obesity [8, 9]. The white adipose tissue (WAT) is the primary component of storing triglycerides. White fat storage is associated with metabolic complications of obesity (such as diabetes) [10]. Energy consumption of the “browning” of white fat influences heat production. The uncoupling protein 1 (UCP1) is the most important marker for predicting heat production [11]. UCP1 is not only an antiobesity protein but also involved in rapid heat production [12]. Peroxisome proliferator-activated receptor-*γ* coactivator-1*α* (Pgc-1*α*) is a transcriptional coactivator that mediates various biological processes related to energy metabolism [13]. It was originally described as a coactivator of peroxisome proliferator-activated receptor-*γ* (PPAR*γ*) that modulated the expression of UCP-1 and thermogenesis in brown fat [14].

Sirtuin 6 (SIRT6), a member of the sirtuin family of NAD-dependent enzymes, exhibits ADP-ribosyl transferase and histone deacetylase activities [15]. SIRT6 plays an important role in various physiological and pathological processes, such as insulin resistance, inflammation, lipid and glucose, and energy metabolism [16]. Studies have shown that the overexpression of SIRT6 in mice prevents metabolic diseases associated with diet-induced obesity [17, 18]. SIRT6 FKO mice are sensitive to obesity induced by an HFD, which is caused by fat cell hypertrophy rather than adipocyte hyperplasia [19]. Meanwhile, activation of SIRT6 may have beneficial effects on glucose and lipid metabolism in obesity and diabetes [20]. SIRT6, functioning as a histone H3 lysine 9 (H3K9) deacetylase, controls the expression of multiple glycolytic genes, as demonstrated [17]. Accordingly, SIRT6 is a potentially novel target to treat obesity.

Chrysophanol is the primary pharmacological active component of *Cassia mimosoides Linn* or *Rhei radix et rhizoma,* which are ancient medicine used for thousands of years in China. Studies have shown that chrysophanol has physiological and pharmacological activities such as lowering blood lipid levels, reducing body weight, preventing liver damage, and as antidiabetic [21–23]. To date, the underlying mechanism of obesity amelioration by chrysophanol remains to be fully elucidated, particularly on brown and beige fat. Therefore, we hypothesize that chrysophanol could affect adipose tissue metabolism, thermogenesis, and insulin tolerance by activating the SIRT6/AMPK pathway in an HFD-induced obese mice model. Additionally, we evaluated chrysophanol's effects, during metabolic disease treatment, dependency on SIRT6 in adipocyte-specific deletion of a SIRT6 mice model.

## 2. Materials and Methods

### 2.1. Animal and Experiment Design

C57BL/6 J mice (male, 6 weeks old) were purchased from the Guangdong Medical Laboratory Animal Center, China. SIRT6^flox/flox^ mice and Adip-Cre mice were purchased from the Jackson Laboratory (USA). SIRT6^flox/flox^ mice were backcrossed with C57BL/6 J mice for at least eight times. To generate mice with SIRT6 FKO, SIRT6^flox/flox^ mice were crossed with Adip-Cre; SIRT6^flox/+^ mice to get Adip-Cre; SIRT6^flox/flox^ mice [24]. Animals were maintained under a constant 12 h light/dark cycle with free access to water and standard chow in a controlled temperature and humidity facility. All animal experimental protocols were approved by the Institutional Animal Care and Use Committee at the institute for Guangzhou University of Chinese Medicine (NO: S 2019025).

Following acclimatization for 1 week, mice were randomly divided into three groups (*n* = 8 for each group) and fed an HFD (number D12492, Changzhou Rat-Mouse II Biotechnology Co., Ltd, China) for 12 weeks to induce obesity. Thereafter, mice were administered an intraperitoneal injection of chrysophanol (6.25 mg/kg/day, 12.5 mg/kg/day). Chrysophanol (>98% pure, C49580, Shanghai, China) was dissolved in the vehicle solution (0.9% NaCl with 1% dimethyl sulfoxide (DMSO) and 1% Tween-80) prior to administration. Following the final administration, mice fasted for 12 h and subsequently anesthetized. Serum and tissue were collected, snap-frozen in liquid nitrogen, and stored at -80°C. BAT was isolated from the interscapular region; the epididymal white adipose tissue (eWAT) was obtained from the epididymis; the inguinal white adipose tissue (iWAT) was isolated from the layer under the skin and outside the abdominal cavity at the hips. Fat mass was the sum of BAT, eWAT, and iWAT.

### 2.2. Cell Culture and Adipocyte Differentiation

3T3-L1 cells were purchased from the American Type Culture Collection (ATCC, Manassas, VA, USA), stored in Dulbecco's Modified Eagle Medium (DMEM), and cultured in a 5% CO_2_ cell incubator at 37°C. Cells incubated until 100% confluency was obtained and contact-inhibited for 2 days. Then, reaching confluence for 2 days, cells were cultured in DMEM which was containing the following components: 50 nM insulin (Sigma, St. Louis, USA), 0.5 mM IBMX (Sigma, USA), and 2 *μ*g/ml dexamethasone (Sigma, USA). Subsequently, the cell differentiation was cultured in DMEM high glucose medium which contained 1 mg/l insulin (Sigma, USA) and 10% fetal bovine serum for another 2 days. Thereafter, cells were cultured in normal DMEM, and the medium was changed every 2 days until differentiation was mature, then treated with different concentrations of chrysophanol (6.25, 12.5, 25, 50, and 100 *μ*M) for 48 h. si-SIRT6 was obtained from General Biol Co., Ltd. (Anhui Province, China). The sequence of si-SIRT6 was 5′-CCAAGUGUAAGACGCAGUATT-3′. 3T3-L1 cells were transfected using Lipofectamine® 2000 (Invitrogen) according to the manufacturer's protocol. The oligonucleotide doses used were the largest doses suggested by the manufacturer's protocols. All transfections were transient; the cells were not harvested for subsequent assays until 48 h posttransfection. Compound C (Cat: HY13418A) was purchased from MedChemExpress (MCE, Monmouth Junction, NJ). Finally, cells were collected for Oil Red O staining and RNA extraction and subsequently used for further analysis.

### 2.3. Cytotoxicity Test Using the Cell Counting Kit-8 (CCK-8) Method

Cell viability was measured using the CCK-8 method. Cells were seeded into 96-well plates containing 100 *μ*L of medium per well. When the cell growth density reached 50%, emodin was administered at different concentrations (6.25, 12.5, 25, 50, and 100 *μ*M, no treatment for the control group) and cultured in an incubator. After 24 h (at 37°C and 5% CO_2_), 10 *μ*L of CCK-8 solution was added to each well at 37°C for 2 h. Thereafter, the absorbance at 450 nm was detected using a microplate reader (Bio-rad iMark-10970, Japan).

### 2.4. Oil Red O Staining

Mature adipocyte cells were stained with Oil Red O to identify differentiation. After washed with phosphate buffered saline (PBS) and fixed with 4% para-formaldehyde for 30 min, adipocytes were then washed twice with PBS, stained with 60% saturated Oil Red O for 10 to 15 minutes, and wash twice with 60% isopropanol as described previously [25]. Finally, adipocytes were imaged using light microscopy.

### 2.5. Glucose Tolerance Test (GTT) and Insulin Tolerance Test (ITT)

During last week of experimentation, a GTT and an ITT were performed following the final chrysophanol treatment [26]. The blood glucose in GTT was performed on male C57BL/6 J mice after overnight fasting for about 16 h. After measuring the level of baseline blood glucose via a tail nick using glucometer, intraperitoneal injection of 1.5 g/kg glucose was administered, and blood glucose concentrations from the tail vein were measured at 0, 15, 30, 60, 90, and 120 min. For the ITT, mice were fasted for 6 h prior to an intraperitoneal injection of 0.75 U/kg human insulin (Lilly), and blood glucose concentrations from the tail vein were measured at 0, 15, 30, 60, 90, and 120 min.

### 2.6. Energy Expenditure Analysis

Energy expenditure was determined using the Comprehensive Lab Animal Monitoring System (CLAMS system, Columbus Instruments) according to the manufacturer's instructions. Mice were acclimated to a metabolic cage system by placing animals in cages for 24 h prior to measurement of oxygen consumption (VO_2_) and carbon dioxide production (VCO_2_) heat production, and spontaneous physical activity. These data were recorded every 5 min for 24 h. Mice were maintained at 22°C under a 12 h light/dark cycle. Food and water were available ad libitum.

### 2.7. Histological Analysis

Tissue fixed in 4% paraformaldehyde was sectioned after being paraffin embedded. Multiple sections were prepared and stained with hematoxylin and eosin for general morphological observations. Finally, images were acquired using an Olympus system and Image J software used to calculate adipocyte size.

### 2.8. Western Blot Analysis

Tissues were homogenized using lysis buffer (keyGEN Bio TECH, China), containing protease and phosphatase inhibitors and centrifuged at 12,000 × *g*, 4°C for 5 min. The supernatant was collected where protein concentration was measured using the BCA Protein Assay kit (keyGEN Bio TECH, China), according to the manufacturer's instructions. The SDS-PAGE protein loading buffer (5×) (Beyotime, China) was added to the sample and heated at 100°C for 5 min to fully denature the protein. Proteins were separated by SDS-PAGE and transferred to a polyvinylidene fluoride membrane and then blocked with 5% BSA in Tris-buffered saline containing 0.1% Tween 20 and incubated overnight at 4°C with primary antibodies targeting phosphorylated hormone sensitive lipase (p-HSL) (ser563) (1 : 1000, #4139, Cell Signaling Technology, USA), hormone sensitive lipase (HSL) (1 : 1000, ab45422, abcam, USA), adenosine 5**'-**monophosphate- (AMP-) activated protein kinase (AMPK) (1 : 1000, ab3760, abcam, USA), and phosphorylated AMPK (p-AMPK) (1 : 1000, ab133448, abcam, USA). Then, the blots were incubated with HRP-conjugated secondary antibodies and detected by Immobilon Western Chemiluminescent HRP Substrate (Millipore, Billerica, MA) using Molecular Imager ChemiDOC™ XBS imaging systems (Bio-Rad Laboratories).

### 2.9. Quantitative Real-Time PCR Analysis

Total RNA was extracted using Trizol reagent (accurate biotechnology CO., LTD, China) according to the manufacturer's instructions and total RNA concentration measured. The E-vo M-ML V Reverse Transcription Kit (Accurate Biotechnology CO., LTD, China) was used to reverse transcribe RNA into cDNA. The real-time PCR kit was ordered from Takara, and the real-time PCR instrument (lightCycler 2.0) was purchased from Roche. A standard curve was drawn based on the Cp value obtained from the reaction and the amplification efficiency of the gene calculated. To analyze the gene expression, real-time quantitative PCR was performed using an ABI Prism 7300 instrument (Applied Biosystems, Foster City, CA). The total reaction volume was 10 *μ*L, which contained 1 *μ*L cDNA. StepOnePlus instrument (Applied Biosystems) was used for PCR amplification. The primer sequences used for amplification of genes are listed in Table 1.

### 2.10. Statistical Analysis

Statistical analysis was performed using two-tailed independent Student's *t*-tests and analysis of variance (ANOVA). *p* values <0.05 were considered statistically significant.

## 3. Results

### 3.1. Chrysophanol Inhibited Lipid Accumulation in 3T3-L1 Adipocytes

**Figure 1:**
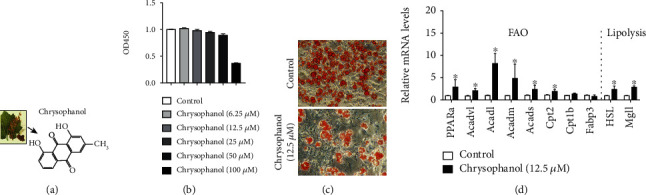
Chrysophanol supplement promoted lipolysis and FAO process in fibroblast 3T3-L1 cell. (a) The chemical formula of chrysophanol. (b) The toxicity of different concentrations of chrysophanol on fibroblast 3 T3-L1 cells was detected by the CCK8 method. (c) Oil Red O staining of 3 T3-L1 cells. (d) The mRNA expression of PPAR*α*, Acadvl, Acadl, Acadm, Acads, Cpt2, Cpt1b, Fabp3, HSL, and Mgll in 3T3-L1 cells was measured by real-time PCR. All data were expressed as the mean ± SD. ^∗^*p* < 0.05 compared with the control group.

The cytotoxic effect of chrysophanol on 3T3-L1 adipocytes was investigated (Figure 1(b)). Chrysophanol at concentrations of 25, 50, and 100 *μ*M showed inhibitory effect on 3T3-L1 cells viability. The viability of 3T3-L1 adipocytes was well kept by chrysophanol at the concentrations of 6.25 and 12.5 *μ*M compared with others, and the excellent protective effect was produced by the compound at the concentration of 12.5 *μ*M. Therefore, pretreatment with chrysophanol at concentrations of 6.25 and 12.5 *μ*M was selected as the conditions of 3T3-L1 adipocytes. Further, investigations were conducted at the specific concentration of 12.5 *μ*M. Next, we investigated the effects of chrysophanol on adipocytes and found that the lipid accumulation of 3T3-L1 adipocytes was significantly decreased following treatment with chrysophanol (Figure 1(c)). HSL and monoacylglycerol lipase (MGLL) can promote the hydrolysis of triglycerides. Acyl-Coenzyme A dehydrogenase very long chain (Acadvl), acyl-CoA dehydrogenase long chain (Acadl), acyl-CoA dehydrogenase medium chain (Acadm), carnitine palmitoyltransferase 2 (Cpt2) ,and carnitine palmitoyltransferase 1b (Cpt1b) participate in lipid and fatty acid metabolism and promote fat breakdown. PPAR*α* is abundant in primary hepatocytes, where it regulates the expression of proteins involved in fatty acid metabolism. Meanwhile, chrysophanol administration increased fatty acid oxidation (FAO) (*PPARα*, *Acadvl*, *Acadl*, *Acadm*, *Cpt2*), lipolysis (*HSL*, *MGLL*), and thermogenesis gene (*Ppargc-1α*, *Prdm16*) expression in 3T3-L1 adipocytes with statistical significance (*p* < 0.05) (Figure 1(d) and Supplementary Figure 5A), but decreased gene expression of adipose marker (*Adipo*, *aP2*), suggesting that chrysophanol promoted lipolysis and restrained lipogenesis to inhibit lipid accumulation.

### 3.2. Chrysophanol Promoted Lipolysis via Activation of the AMPK Pathway to Alleviate Obesity and Fat Accumulation in HFD-Induced Obese Mice

**Figure 2:**
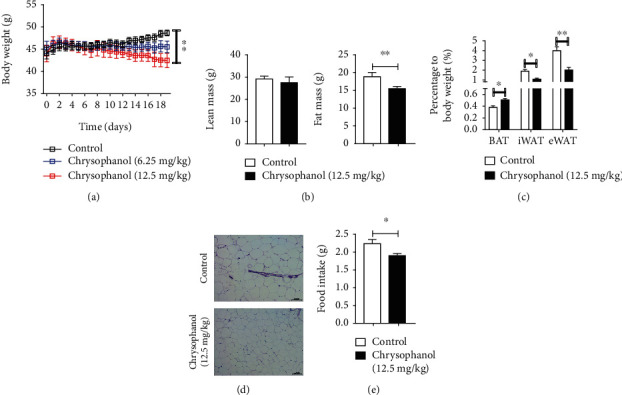
Chrysophanol protected mice against HFD-induced obesity. (a) Body weight of mice (20- to 22-week-old) fed with HFD for 18 d. (b) Fat mass, lean mass, (c) BAT, iWAT, and eWAT percentage to body weight. (d) H&E staining of WAT sections. Scale bar, 100 *μ*m, and (e) food intake of HFD-induced obese mice treated with or without chrysophanol. All data were expressed as the mean ± SD. ^∗^*p* < 0.05 and ^∗∗^*p* < 0.01 compared with the control group (*n* = 8).

To study the effects of chrysophanol on obesity, we measured mice's body weight and food intake. As shown in Figure 2(a), chrysophanol significantly reduced the weight of HFD-induced obese mice. Meanwhile, the mice food intake was significantly reduced by chrysophanol intervention (Figure 2(e)). To further investigate the mechanism of chrysophanol in alleviating obesity, the adipose tissue proportion and morphology were analyzed. We found that chrysophanol significantly decreased fat mass, especially the weight of iWAT and eWAT, but increased BAT in HFD-induced obese mice (Figures 2(b) and 2(c), Supplementary Figure 1). Following chrysophanol treatment, adipocyte volume in HFD-induced obese mice was consistently smaller (Figure 2(d)).

**Figure 3:**
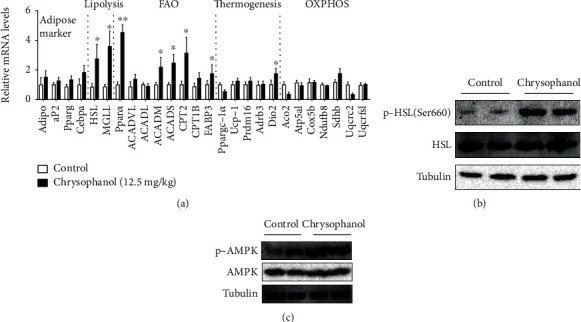
Chrysophanol regulated FAO and lipolysis in iWAT of HFD-induced obese mice. (a) The expression of adipose maker, lipolysis, FAO, thermogenesis, and oxidative phosphorylation (OXPHOS) relative mRNA in the iWAT of HFD-induced obese mice was measured by real-time quantitative PCR. (b, c) The p-HSL (Ser660), HSL, AMPK, and p-AMPK protein levels in the iWAT of HFD-induced obese mice were determined by Western blotting. All data are expressed as the mean ± SD. ^∗^*p* < 0.05, ^∗∗^*p* < 0.01 compared with the control group. Mice treated with a 12.5 mg/kg dose of chrysophanol (*n* = 8).

To explore whether the effects of chrysophanol on obesity were related to energy or metabolism, the related gene expression in the adipose tissue was analyzed. Several lipolysis factors, genes of lipolysis and FAO in WAT and thermogenic genes in BAT, were significantly increased following chrysophanol treatment (*p* < 0.05) (Figure 3(a) and Supplementary Figure 2). Lipolysis is primarily regulated by three hydrolases: adipose triglyceride lipase (ATGL), HSL, and MGLL [27] and is also regulated by perilipin (PLIN), cyclic AMP-Protein Kinase A (cAMP-PKA) [28], extracellular regulated protein kinases 1/2 (ERK1/2) [29], AMPK [27], and other signaling pathways. Studies have shown that the HFD can downregulate the expression of HSL and inhibit the activation of AMPK [30]. In Figure 3(a), the mRNA expression of HSL and MGLL in the chrysophanol group increased, as well as the expression of HSL and p-HSL (Ser660) protein (Figure 3(b)), which indicated that chrysophanol promoted lipolysis by activating HSL through phosphorylation at Ser660. Meanwhile, the protein expression of AMPK and p-AMPK of the chrysophanol group increased (Figure 3(c)). These data indicated that chrysophanol phosphorylated AMPK, activated AMPK, and promoted lipolysis in WAT. These results suggested that chrysophanol promoted the lipolysis and alleviate obesity by activating AMPK and HSL. To investigate how the AMPK pathway works in lipid metabolism, Compound C, an AMPK inhibitor, was used on 3T3-L1 cells, and found that it attenuated chrysophanol inhibition of lipid accumulation (Supplementary Figure 4 and Supplementary Figure 5). These suggested that chrysophanol effected through the AMPK pathway.

### 3.3. Chrysophanol Induced Thermogenesis in HFD-Induced Obese Mice

**Figure 4:**
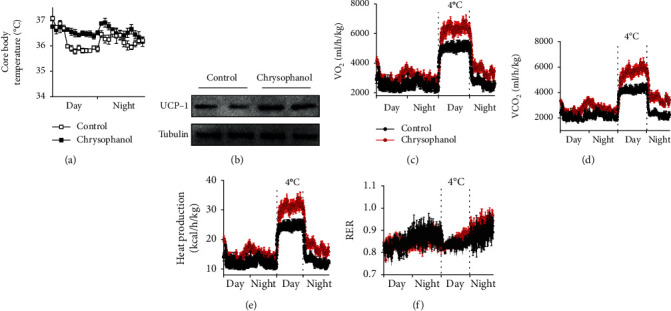
Chrysophanol increased thermogenesis in HFD-induced obese mice. (a) Curves of 24 h core body temperature of HFD-induced obese mice in both groups. (b) The UCP-1 in BAT protein levels in iWAT of HFD-induced obese mice was determined by Western blotting. (c–f) Visual comparisons and 24 h O_2_ consumption, CO_2_ production, heat production, and RER dynamic variations of HFD mice and red curves represented HFD-induced obese mice treated with chrysophanol, and black curves represented those without chrysophanol. Data represented the mean ± SD of two independent experiments. Mice treated with a 12.5 mg/kg dose of chrysophanol (*n* = 8).

To identify the mechanism by which chrysophanol ameliorated thermogenesis, we systematically analyzed the effect of chrysophanol on energy expenditure using metabolic cage to comprehensively monitor lab animals. The data showed that HFD-induced obese mice in the chrysophanol group have higher oxygen consumption and carbon dioxide production (Figures 4(c) and 4(d)). In addition, mice in the chrysophanol group exhibited an evident increase in core body temperature and heat production in 24 h (Figures 4(a) and 4(e)). However, there was no difference in the respiratory exchange rate (RER) between both groups. Therefore, during the cold tolerance tests (at 4°C), chrysophanol significantly promoted cold-induced thermogenesis by greater oxygen consumption, carbon dioxide, and heat production (*p* < 0.05) (Figures 4(c)–4(e)). *UCP-1*, a brown fat-specific thermogenic gene, regulates thermogenesis and energy metabolism to maintain body energy homeostasis. Chrysophanol increased the protein expression of UCP-1 in BAT of HFD-induced obese mice (Figure 4(b)). These results suggested that chrysophanol enhanced thermogenesis and adaptive thermogenesis in HFD-induced obese mice.

### 3.4. Chrysophanol Improved Glucose Tolerance and Insulin Sensitivity of HFD-Induced Obese Mice

**Figure 5:**
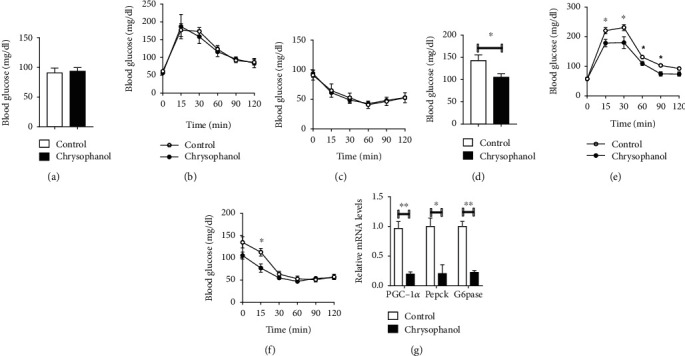
Chrysophanol decreased FBG and promoted glucose tolerance and insulin sensitivity in HFD-induced obese mice. (a) FBG, (b) GTT, and (c) ITT of normal mice. (d) FBG, (e) GTT, and (f) ITT of HFD-induced obese mice. (g) The expression of PGC-1*α* and glycogen-related enzyme (Pepck and G6pase) in the liver of HFD-induced obese mice was measured by real-time PCR. All data were expressed as the mean ± SD, ^∗^*p* < 0.05 and ^∗∗^*p* < 0.01 compared with the control group. chrysophanol, mice treated with a 12.5 mg/kg dose of chrysophanol (*n* = 8).

Glucose metabolism is closely correlated with obesity. GTT and ITT were used to assess the effect of chrysophanol in glucose metabolism. We found that chrysophanol had improved tolerance to glucose load and was more sensitive to insulin in HFD-induced obese mice, but not in normal mice. (Figures 5(a)–5(f)). Pgc-1*α* influences energy metabolism regulation, including sugar uptake, gluconeogenesis, and insulin secretion. Phosphoenolpyruvate carboxykinase (PEPCK) is a key enzyme in stimulating glucose production. Glucose-6-phosphatase (G6Pase) is a multicomponent enzyme system that hydrolyzes glucose-6-phosphate in gluconeogenesis and glycogenolysis. Moreover, chrysophanol treatment in the liver of HFD-induced obese mice can significantly suppressed gluconeogenesis genes, including *Pgc1α* (*p* < 0.01), *Pepck* (*p* < 0.05), and *G6pase* (*p* < 0.01) levels in liver (Figure 5(g)). These results suggested that chrysophanol improved glucose tolerance and insulin sensitivity of obese mice, rather than nonobese mice.

### 3.5. SIRT6 Inhibitors and Si-SIRT6 Limited the Inhibitory Effect of Chrysophanol on Lipid Accumulation in 3T3-L1 Adipocytes

**Figure 6:**
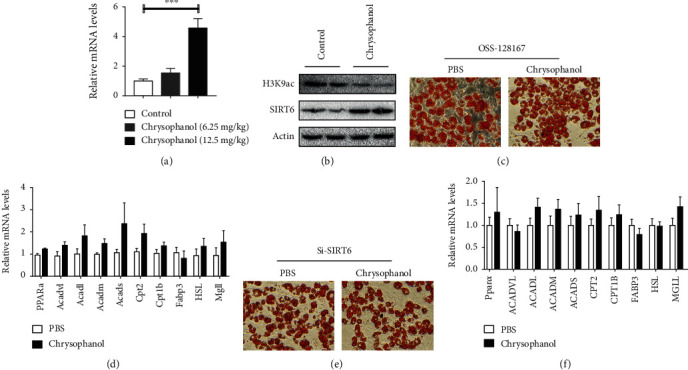
SIRT6 inhibitors attenuated the effect of chrysophanol in promoting FAO and lipolysis. (a) SIRT6 mRNA detection in BAT of mice treated with different doses of chrysophanol (6.25 mg/kg and 12.5 mg/kg). (b) The protein expression of H3K9ac and SIRT6 in BAT were determined by Western blotting. (c) Oil Red O staining of 3 T3-L1cells in both groups using the SIRT6 inhibitor, OSS-128167. (d) Lipolysis and FAO relative mRNA expression in 3 T3-L1cells under cotreatment of chrysophanol with OSS-128167. (e) Oil Red O staining of 3 T3-L1 cells in both groups using Si-SIRT6. (f) Lipolysis and FAO relative mRNA expression in 3T3-L1cells under cotreatment of chrysophanol with Si-SIRT6. All data are expressed as the mean ± SD. ^∗∗∗^*p* < 0.001 compared with the control group (*n* = 8).

Some studies indicated that the liver-specific SIRT6 expression is decreased in rats with spontaneous obesity and metabolic syndrome [31]. SIRT6 can deacetylate histone hyperacetylated H3K9 (H3K9ac) and hyperacetylated H3K56 (H3K56Ac) [32]. As is shown in Figure 6(a), chrysophanol significantly increased the mRNA expression of SIRT6 in obese mice in a dose-dependent manner (*p* < 0.001). Consistently, chrysophanol increased the SIRT6 expression in adipocytes (Figure 6(b)). In contrast, chrysophanol decreased the H3K9ac expression in BAT of HFD-induced obese mice (Figure 6(b)). To further understand the chrysophanol undelaying mechanism of action, SIRT6 inhibitor, OSS-128167, and si-SIRT6 were added to 3T3-L1 adipocytes. No significant reduction in droplet size in cells treated with SIRT6 inhibitor (OSS-128167) or si-SIRT6 was observed after using chrysophanol (Figure 6(c) and Figure 6(e)), similar to the control group (Figure 1(c)). Moreover, SIRT6 inhibitor and si-SIRT6 both abrogated the effect of chrysophanol in increasing the mRNA expression of FAO and lipolysis genes (Figure 6(d) and Figure 6(f)). Following with si-SIRT6 treatment, chrysophanol failed to upregulate the expression of main genes of thermogenesis (Supplementary Figure 3).

### 3.6. Adipocyte-Specific Deletion of SIRT6 Abrogated the Effects of Chrysophanol in Improving Obesity, Thermogenesis, and Insulin Sensitivity

**Figure 7:**
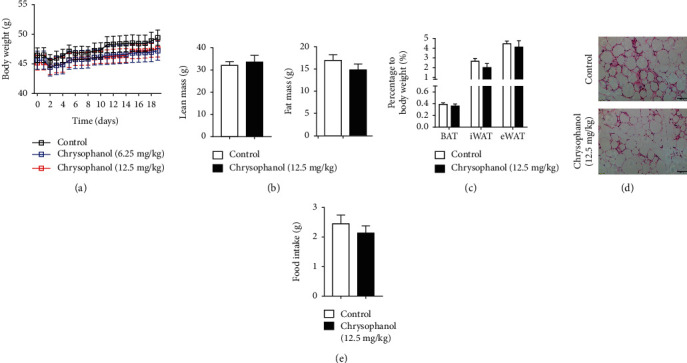
Adipocyte-specific deletion of SIRT6 attenuated the protective effect of chrysophanol on HFD-induced obese mice. WT and SIRT6 FKO mice experimental design as described under materials and methods. (a) Body weight of WT and SIRT6 FKO mice (20- to 22-week-old) fed with HFD for 18d. (b) Fat mass, lean mass, (c) BAT, iWAT, and eWAT percentage to body weight. (d) H&E staining of WAT. Scale bar, 100 *μ*m, and (e) food intake of HFD-induced obese mice treated with or without chrysophanol. All data were expressed as the mean ± SD (*n* = 8).

**Figure 8:**
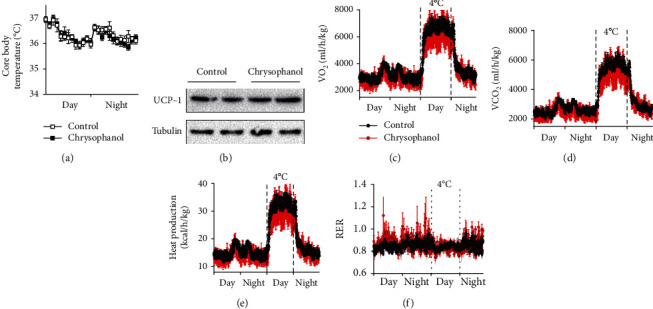
Adipocyte-specific deletion of SIRT6 attenuated the role of chrysophanol in increasing thermogenesis in HFD-induced obese mice. WT and SIRT6 FKO mice experimental design as described under materials and methods. (a) Curves of 24 h core body temperature of HFD mice in both groups. (b) The protein expression of UCP-1 in BAT of HFD-induced obese mice were determined by Western blotting. (c–f) Visual comparisons and 24 h O_2_ consumption, CO_2_ production, heat production, and RER dynamic variations of HFD mice and red curves represented HFD-induced obese mice treated with chrysophanol, and the black curves represent those without chrysophanol. Data represented the mean ± SD of two independent experiments (*n* = 8).

To verify whether the SIRT6 gene plays a role in the pharmacological action of chrysophanol, we conducted experiments using SIRT6 FKO mice. These involved changes in the body weight and fat volume, expression of relative genes in BAT, thermogenesis, glucose metabolism, and insulin resistance. We found that the SIRT6 FKO prevented the ability of chrysophanol to reduce body weight, fat mass, and food intake of HFD-induced obese mice (Figures 7(a), 7(b), and 7(d)).There was no difference between both groups in iWAT/eWAT and BAT percentages (Figure 7(c)) and adipocyte size in iWAT (Figure 7(d)). The effect in upregulating of thermogenesis genes by chrysophanol was restrained (Supplementary Figure 2 and Supplementary Figure 6). We then found that mice in chrysophanol and control groups showed similar oxygen consumption and carbon dioxide production in SIRT6 FKO obese mice (Figures 8(c) and 8(d)), and that it did not exhibit an evident increase in core body temperature and heat production in 24 h (Figures 8(a) and 8(e)). In addition, there was no significant difference in the RER between both groups. Furthermore, cold tolerance tests (at 4°C) showed that chrysophanol did not promote oxygen consumption, carbon dioxide, and heat production in SIRT6 FKO obese mice (Figures 8(c)–8(e)). Meanwhile, the absence of adipocyte-specific SIRT6 suppressed the role of chrysophanol in increasing the UCP-1 expression in BAT (Figure 8(b)). These results suggested that the effect of chrysophanol on obesity and thermogenesis partially depend on the SIRT6 gene.

**Figure 9:**
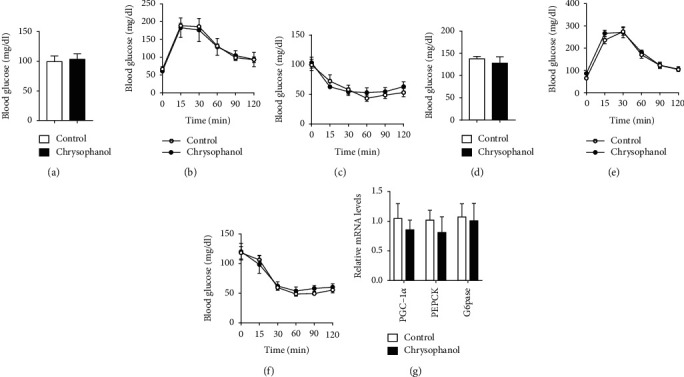
Adipocyte-specific deletion of SIRT6 attenuated the role of chrysophanol in promoting FBG, glucose tolerance, and insulin sensitivity in HFD-induced obese mice. Mice with SIRT6 FKO as described under materials and methods. (a) FBG, (b) GTT, and (c) ITT of normal diet mice. (d) FBG, (e) GTT, and (f) ITT of HFD-induced obese mice. (g) The expression of PGC-1*α* and glycogen-related enzyme (Pepck and G6pase) in the liver tissue of HFD mice was measured by real-time PCR. All data were expressed as the mean ± SD (*n* = 8).

Fasting blood-glucose (FBG), GGT, and ITT in both groups were dynamically measured on SIRT6 FKO obese mice (Figures 9(a)–9(f)). We found no significant difference between both groups in FBG, GGT, and ITT of normal mice (Figures 9(a)–9(f)). The mRNA expression of gluconeogenesis genes involving PGC-1*α*, Pepck, and G6pase of the liver had been slightly suppressed with chrysophanol treatment but had no statistical significance (*p* > 0.05) (Figure 9(g)). Notably, the beneficial effects of chrysophanol on reducing FBG, GGT, and ITT were largely diminished in SIRT6 FKO obese mice, suggesting a critical role of SIRT6 in mediating the effects of chrysophanol in preventing obesity, improving glucose tolerance and insulin sensitivity.

## 4. Discussion

When long-term energy intake exceeds energy consumption, storage in the fat tissue in the form of triglycerides increases, causing obesity, which has currently become one of the most serious public health problems worldwide [33]. In terms of treatment, long-term dieting and exercise are often difficult to maintain. A large percentage of patients fail and revert to their weight before the weight loss plan was implemented, 1 year following program completion [34]. A mouse model of obesity induced by an HFD has been used to study obesity-related diseases, which are associated with fat accumulation, thermogenesis, insulin sensitivity, and glucose tolerance. However, treatment with chrysophanol could considerably reduce the regeneration of adipose cells in vitro, as well as body weight of HFD-induced obese mice, effectively inhibiting lipid accumulation. Based on this, we further studied the mechanism of chrysophanol-mediated obesity and fatty liver disease through its effect on thermogenesis, insulin sensitivity, glucose tolerance, and regulation of mRNA and protein expression.

Adipose tissues store energy, where excess carbohydrates and fatty acids are stored in the form of lipids. WAT has a high capacity to store triglycerides, whereas BAT is rich in mitochondria and therefor has a lipolytic function and can burn a high amount of energy using UCP-1 [35]. In our study, treatment with chrysophanol not only reduces body weight and blood sugar in mice but also induces heat production by turning WAT into BAT, thus inhibiting lipogenesis. In addition, chrysophanol can improve insulin sensitivity in HFD-induced obese mice [23]. Overall, this demonstrates the important role of chrysophanol in maintaining glycolipid homeostasis.

SIRT6, regulates the acetylation of histone H3K9, effects the stability of telomere, transcriptional regulation, and DNA damage response [36–38]. In a recent study, it was confirmed that the deacetylation of the X-box binding protein 1 (XBP1s) by SIRT6 can alleviate hepatic steatosis caused by endoplasmic reticulum stress [39]. Yao et al. [24] found that the expression level of SIRT6 in BAT was significantly higher than that in WAT, and the expression of SIRT6 in BAT of obese mice significantly decreased. SIRT6 is induced through the phosphorylation and of activation of transcription factor 2 (ATF2) to enhance the expression of PGC-1*α* by recruiting phosphorylated ATF2 to its promoter region. Moreover, the overexpression of SIRT6 promotes the expression of thermogenic genes and mitochondrial respiration. However, deletion of SIRT6 in the adipose tissue markedly impairs the function of thermogenesis of BAT, causing substantial “whitening” of BAT in mice, while mice's oxygen consumption rate and body temperature decrease, leading to obesity. SIRT6 knockout mice develop severe hypoglycemia and other defects causing death [40]. To the best of our knowledge, this study is the first to suggest that chrysophanol promoted thermogenesis in a SIRT6-dependent manner by activating the AMPK signaling pathway in BAT, reducing body weight of HFD-induced obese mice. Interestingly, when treated with an SIRT6 inhibitor, the effects of reducing lipid deposition, FAO, and lipolytic gene overexpression were eliminated. Therefore, we suspect that chrysophanol is likely to function through SIRT6. Meanwhile, the results of specific knockout of SIRT6 in mice support our results. In HFD-induced obese mice with SIRT6 FKO, chrysophanol's ability to reverse the reduction of heat production is inhibited increasing mice's body weight. These results may suggest that chrysophanol regulates heat production and reduces obesity through SIRT6. Chrysophanol and targeted SIRT6 therapy have broad prospects for the treatment of metabolic ailments such as obesity, type 2 diabetes, and fatty liver disease.

AMPK, an AMP-dependent protein kinase, is a cellular energy sensor. Previous studies have shown that it can promote fatty acid metabolism and mitochondrial biogenesis, which help to induce the catabolic pathway and production of adenosine triphosphate (ATP) in nutrient deprivation environments [41]. Furthermore, AMPK helps to enhance utilization of glucose by skeletal muscles and ameliorates insulin sensitivity [42, 43], inhibits adipose regeneration, and fortifies lipolysis and lipid burning of adipose cells [44, 45]. Wang et al. [46] had indicated that the phosphodiesterase 4 inhibitor (PDE4), rolipram, can promote AMPK phosphorylation and upregulate the expression level of SIRT6 in the liver and kidney of aging mice. This had a positive effect on lipid metabolism by inhibiting adipose deposition associated with senescence, through increasing the concentration of intracellular cyclic adenosine monophosphate (cAMP). Similarly, Fan, Y. et al. [47] found that SIRT6 plasmid transfection can reverse the reduction, deformation, and apoptosis of podocyte mitochondria caused by high glucose, as well as the low expression of SIRT6. Moreover, it increases AMPK phosphorylation, reduce reactive oxygen species (ROS), and alleviate oxidative stress. Recent experiments have also confirmed the importance of AMPK in maintaining mitochondrial homeostasis [48]. Chrysophanol markedly upregulated rRNA levels of MGLL and HSL, which are key lipolysis enzymes. It also upregulated *β*-oxidation-associated gene expression in fatty acids, thus increasing WAT breakdown. Fatty acids are substrates and activators for BAT production, which increase the UCP1 and PGC-1*α* expression and further activate BAT's. Conversely, the deacetylase activity of SIRT6 depends on the presence of free fatty acid (FFA) [49]. The expression of SIRT6, AMPK, and p-AMPK in mice treated with chrysophanol formed a beneficial cycle, and fatty acids were transported to BAT for oxidation and heat production.

UCP1 uniquely expresses in BAT, where no shivering thermogenesis is achieved through it [35]. The thermogenic activity of BAT is regulated by the sympathetic and nonsympathetic nervous system, *β*3-adrenergic receptor agonist, neuropeptide, and various other materials that may be increased by stimulating the activity of the former [50, 51]. However, iris pigment and natriuretic peptides can induce the thermogenic gene expression such as UCP1 and PGC-1*α*, which can directly increase its activity [14, 52]. However, SIRT6 acetylates PGC-1*α* in hepatocytes, thereby inhibiting the gluconeogenesis pathway, which in primarily activated by the General Control Nondepressed Protein 5 [53]. The PGC-1*α* expression in mice liver treated with chrysophanol was low. The Pepck and G6pase expression, which are closely associated with gluconeogenesis, were also significantly reduced (Pgc1*α* (*p* < 0.01), Pepck (*p* < 0.05), and G6pase (*p* < 0.01)). This consequently reduced hepatic gluconeogenesis and reduced conversion into adipose storage, which ultimately helps to mitigate obesity.

Altogether, our study has demonstrated that chrysophanol can inhibit lipid accumulation and prevent obesity by activating the brown adipocyte SIRT6/AMPK signaling pathway, increasing adipose decomposition, FAO, improving insulin sensitivity and thermogenesis in HFD-induced obese mice. Chrysophanol can upregulate the expression of SIRT6, AMPK, and UCP-1, activating downstream signals of the nonsympathetic nervous system to increase thermogenesis. Whether chrysophanol increases heat production through other pathways, like the sympathetic nervous system in excited obese mice, remains to be elucidated. Chrysophanol is effective in treating obesity without serious adverse events being observed with the dose used. Chrysophanol and targeted SIRT6 therapy have broad prospects for the treatment of metabolic diseases.

## Figures and Tables

**Table 1:** Primer information for gene amplification.

Primer	Sequences
UCP1	F1: 5'-CTGCCAGGACAGTACCCAAG-3'
	R1: 5'-TCGTGGTCTCCCAGCATAGA-3'
Adipo	F1: 5'-GAAGTTGATTATATGAATCAGG-3'
	R1: 5'-GAAGTTGATTATATGAATCAGA-3'
AP2	F1: 5'-TTTGTATCACCCTAACCTG-3'
	R1: 5'-TGCCCTTTCGTAAACTCT-3'
Pparg	F1: 5'-TCAAGAAGACGGAGACAGACA-3'
	R1: 5'-TGGAAGAAGGGAAATGTTGG-3'
Cebpa	F1: 5'-CTTCAACGACGAGTTCCTGGCCGA-3'
	R1: 5'-AGCTGCTTGGCTTCATCCTCCT-3'
HSL	F1: 5'-GCTGGGCTGTCAAGCACTGT-3'
	R1: 5'-GTAACTGGGTAGGCTGCCAT-3'
Mgll	F1: 5'-GACGGACAGTACCTCTTT-3'
	R1: 5'-AGAAAAGTAGGTTGGCCTCT-3'
Ppar*α*	F1: 5'-CGGCGTTGAAAACAAGGAGG-3'
	R1: 5'-CCTTGGCAAATTCCGTGAGC-3'
Acadvl	F1: 5'-CTCAGTGAAGAACAGGCA-3'
	R1: 5'-CTTGGCAGGGTCATTCACTT-3'
Acadl	F1: 5'-ATCTTTTCCTCGGAGCATG-3'
	R1: 5'-TTTCTCTGCGATGTTGATGC-3'
Acadm	F1: 5'-AACACAACACTCGAAAGC-3'
	R1: 5'-TTCTGCTGTTCCGTCAACTCA-3'
Acads	F1: 5'-TGACTTTGCCGAGAAGGA-3'
	R1: 5'-ACTCAGCTCCTCTGGCACAT-3'
Cpt2	F1: 5'-CAAAAGACTCATCCGCTT-3'
	R1: 5'-CATCACGACTGGGTTTGGGT-3'
Cpt1b	F1: 5'-AAGAGACCCCGTAGCCAT-3'
	R1: 5'-GACCCAAAACAGTATCCCAAT-3'
Fabp3	F1: 5'-ACCAAGCCTACTACCATC-3'
	R1: 5'-CCTCGTCGAACTCTATTCCCA-3'
Ppargc-1*α*	F1: 5'-GGAGCTCCAAGACTCTAG-3'
	R1: 5'-CCAAAGTCTCTCTCAGGTAGC-3'
Prdm16	F1: 5'-CAGCACGGTGAAGCCATT-3'
	R1: 5'-GCGTGCATCCGCTTGTG-3'
Adrb3	F1: 5'-CCTTGGGCGAAACTGGTT-3'
	R1: 5'-GTTGGTGACAGCTAGGTAGC-3'
Dio2	F1: 5'-CAGTGTGGTGCACGTCTC-3'
	R1: 5'-TGAACCAAAGTTGACCACCA-3'
Aco2	F1: 5'-ATCGAGCGGGGAAAGAC-3'
	R1: 5'-TGATGGTACAGCCACCTTAG-3'
Atp5a1	F1: 5'-TCTCCATGCCTCTAACACT-3'
	R1: 5'-CCAGGTCAACAGACGTGTCA-'3
Cox5b	F1: 5'-GCTGCATCTGTGAAGAGG-3'
	R1: 5'-CAGCTTGTAATGGGTTCCACA-3'
Ndufb8	F1: 5'-TGTTGCCGGGGTCATATC-3'
	R1: 5'-AGCATCGGGTAGTCGCCATA-3'
Sdhb	F1: 5'-CTGAATAAGTGCGGACCT-3'
	R1: 5'-AGTATTGCCTCCGTTGATGTT-3'
Uqcrc2	F1: 5'-AAAGTTGCCCCGAAGGTT-3'
	R1: 5'-GAGCATAGTTTTCCAGAGAA-3'
Uqcrfsl	F1: 5'-GGTAACTGCAACTACTAC-3'
	R1: 5'-CTTGATCTCGATCTTCGACAT-3'
*β*-Actin	F1: 5'-TCCCTGGAGAAGAGCT-3'
	R1: 5'-AGCACTGTGTTGGCGTACAG-3'

## Data Availability

The data used to support the findings of this study are available from the corresponding author upon request.

## Abbreviations

HFD: High-fat diet
SIRT6: Sirtuin-6
FKO: Fat-specific SIRT6 knockout
UCP1: Uncoupling protein 1
AMP: Adenosine 5′-monophosphate
AMPK: AMP-activated protein kinase
BAT: Brown adipose tissue
WAT: White adipose tissue
PPAR*γ*: Peroxisome proliferator-activated receptor *γ*
PGC1*α*: PPAR*γ* coactivator-1*α*
H3K9: Histone H3 lysine 9
eWAT: Epididymal white adipose tissue
iWAT: Inguinal white adipose tissue
GTT: Glucose tolerance test
ITT: Insulin tolerance test
VO_2_: Oxygen consumption
VCO_2_: Carbon dioxide production
FBG: Fasting blood glucose
PPAR*γ*: Peroxisome proliferator-activated receptor *γ*
WAT: White adipose tissue
ATF2: Activating transcription factor 2
MGLL: Monoacylglycerol lipase
HSL: Hormone-sensitive lipase
p-HSL: Phosphorylation of HSL
FAO: Fatty acid oxidation
CCK8: Cytotoxicity test using the cell counting Kit-8
PEPCK: Phosphoenolpyruvate carboxykinase
G6pase: Glucose-6-phosphatase
Acadvl: Acyl-coenzyme a dehydrogenase very long chain
Acadl: Acyl-CoA dehydrogenase long chain
Acadm: Acyl-CoA dehydrogenase medium chain
Cpt2: Carnitine palmitoyltransferase 2
Cpt1b: Carnitine palmitoyltransferase 1b
RER: Respiratory exchange rate
WT: Wild type.

## Supplementary Materials

Supplementary Materials Supplementary Figure 1: chrysophanol regulated the mRNA expression of adipose maker and in 3 T3-L1 adipocytes. Supplementary Figure 2: chrysophanol increased absolute weight of BAT and decreased that one's of WAT weight of HFD-induced obese mice. Supplementary Figure 3: chrysophanol regulated thermogenesis in BAT of HFD-induced obese mice. Supplementary Figure 4: silencing SIRT6 in adipocytes attenuated the induction effect of chrysophanol in the expression of thermogenic genes. Supplementary Figure 5: AMPK inhibitors attenuated the inhibitory effect of chrysophanol on adipose accumulation. Supplementary Figure 6: AMPK inhibitors attenuated the regulatory effect of chrysophanol on adipose markers and thermogenic-related gene expression. Supplementary Figure 7: the effect of chrysophanol in promoting thermogenesis was blunted in BAT of SRIT6 FKO mice.
