# Supplementary material for: Chrysophanol Alleviates Metabolic Syndrome by Activating the SIRT6/AMPK Signaling Pathway in Brown Adipocytes
Source: Oxid Med Cell Longev. 2020 Nov 12;2020:7374086. doi: 10.1155/2020/7374086 (PMC7683138; doi:10.1155/2020/7374086)
Supplement: Supplementary Materials — Supplementary Figure 1: chrysophanol regulated the mRNA expression of adipose maker and in 3 T3-L1 adipocytes. Supplementary Figure 2: chrysophanol increased absolute weight of BAT and decreased that one's of WAT weight of HFD-induced obese mice. Supplementary Figure 3: chrysophanol regulated thermogenesis in BAT of HFD-induced obese mice. Supplementary Figure 4: silencing SIRT6 in adipocytes attenuated the induction effect of chrysophanol in the expression of thermogenic genes. Supplementary Figure 5: AMPK inhibitors attenuated the inhibitory effect of chrysophanol on adipose accumulation. Supplementary Figure 6: AMPK inhibitors attenuated the regulatory effect of chrysophanol on adipose markers and thermogenic-related gene expression. Supplementary Figure 7: the effect of chrysophanol in promoting thermogenesis was blunted in BAT of SRIT6 FKO mice. [file 7374086.f1.doc]

**Supplementary data**


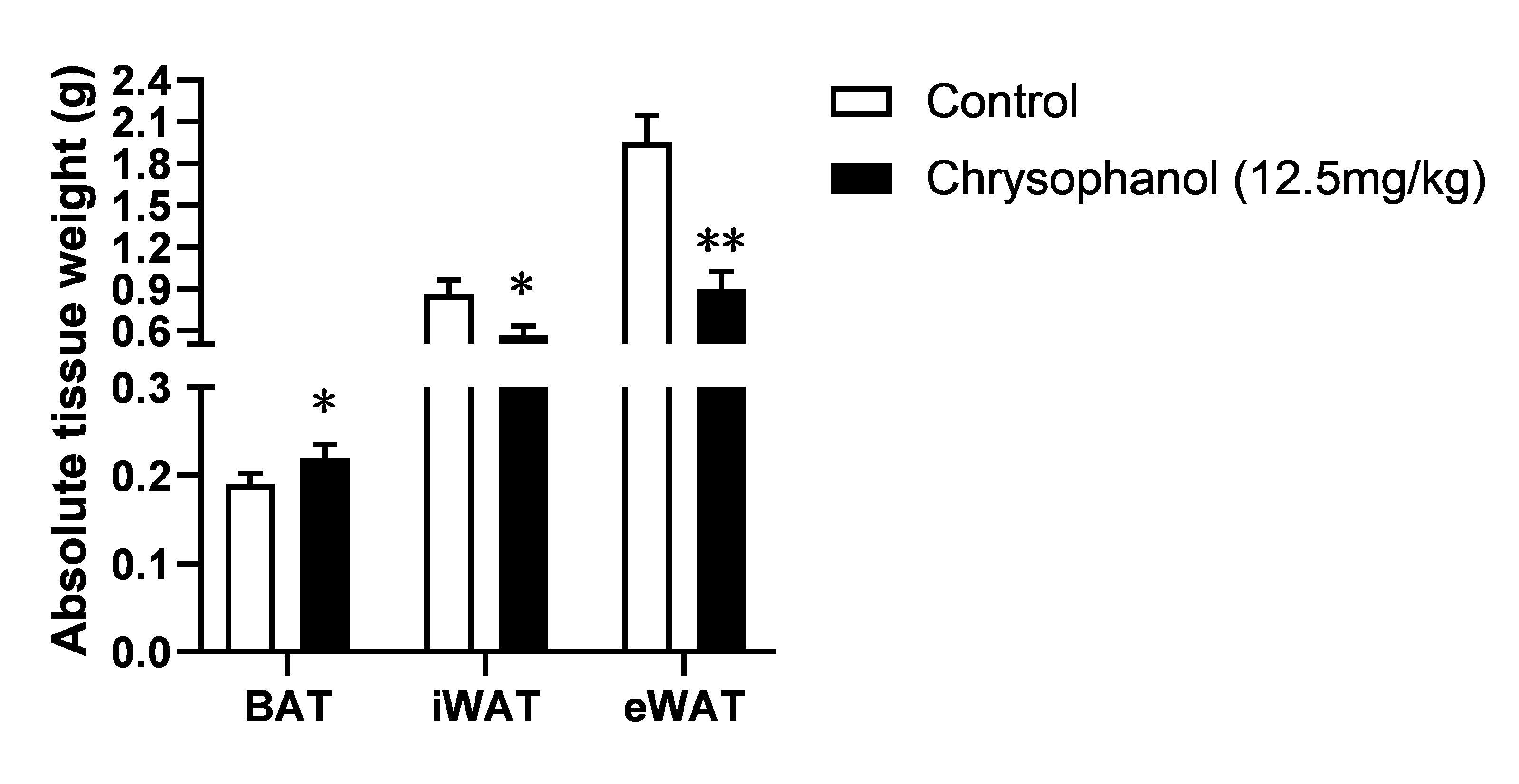


**Supplementary Figure 1: Chrysophanol increased absolute weight of BAT and decreased that one’s of WAT weight of HFD-induced obese mice.** Absolute weight of BAT, iWAT, eWAT of HFD mice in Chrysophanol and control group. All data were expressed as the mean ± SD. **p* < 0.05, ***p* < 0.01 compared with the control group (n = 8).


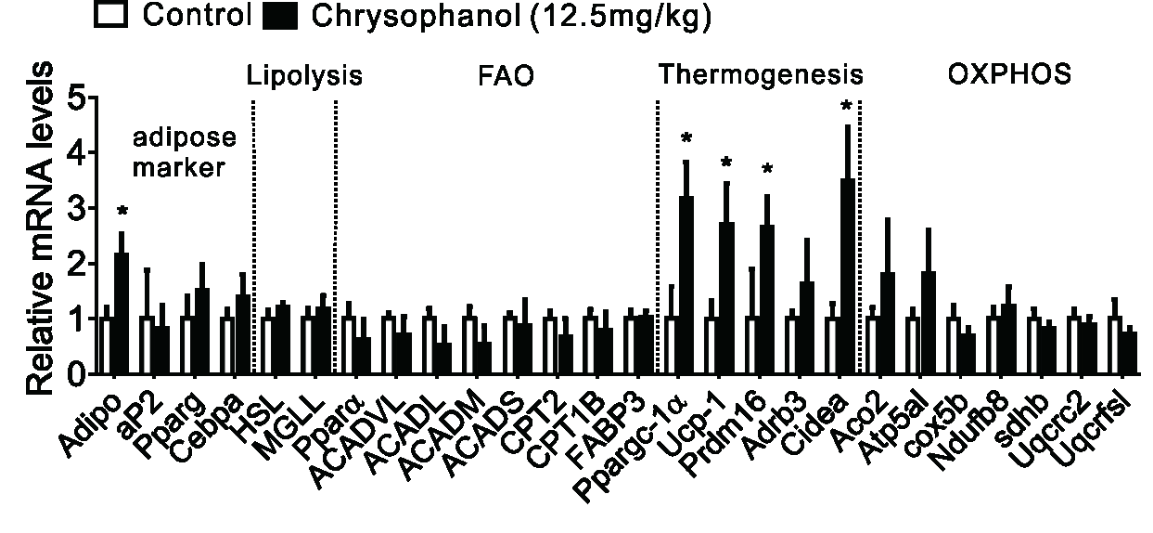


**Supplementary Figure 2: Chrysophanol regulated thermogenesis in BAT of HFD-induced obese mice.** The mRNA expression of adipose markers, lipolysis, FAO, thermogenesis, and OXPHOS in BAT of HFD-induced obese mice was measured by real-time PCR. All data were expressed as the mean ± SD. **p* < 0.05 compared with the control group (n=8).


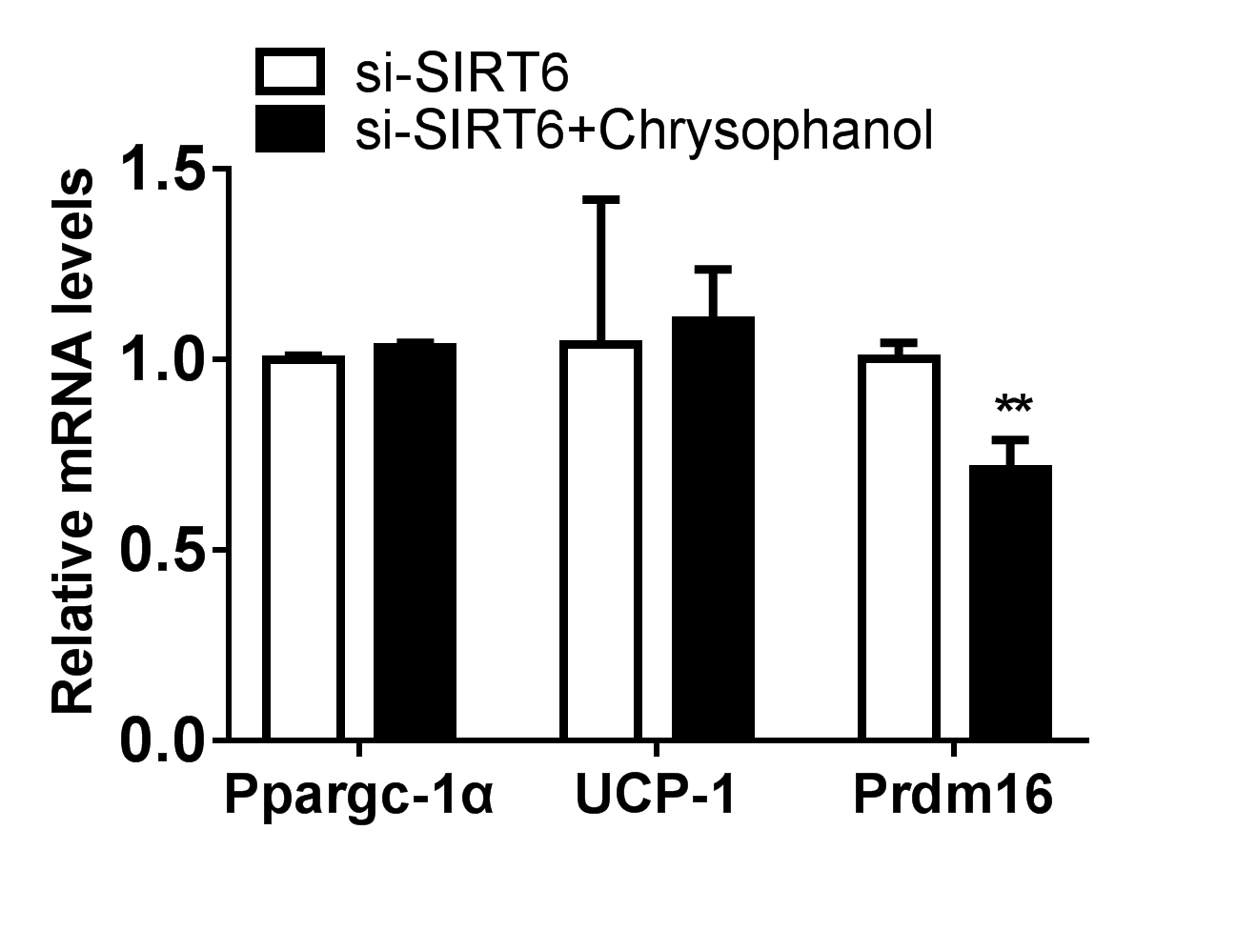


**Supplementary Figure 3: Silencing SIRT6 in adipocytes attenuated the induction effect of Chrysophanol in expression of thermogenic genes.** The mRNA expression of Ppargc-1α, UCP-1, Pradm16 in adipocytes was measured by real-time PCR. Adipocytes treated with PBS or Chrysophanol (12.5 μM). All data were expressed as the mean ± SEM. ***p* < 0.01 compared with the control group (n=3).


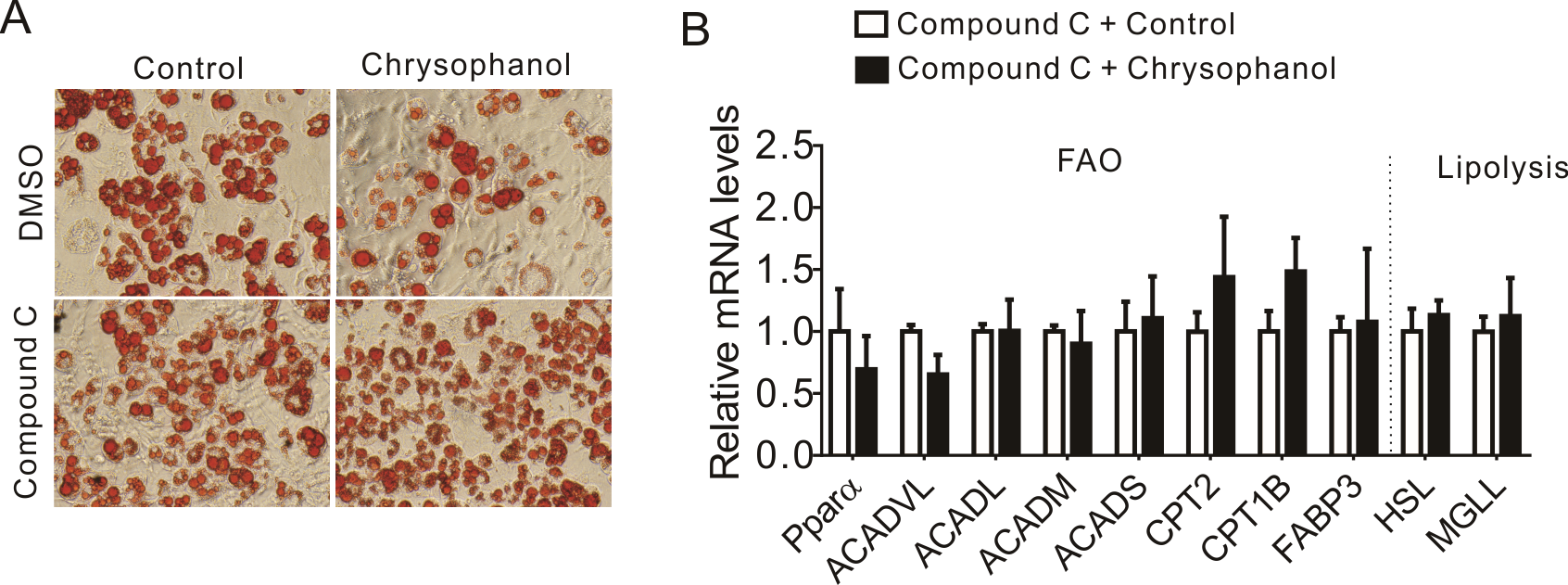


**Supplementary Figure 4: AMPK inhibitors attenuated the inhibitory effect of Chrysophanol on adipose accumulation. (A)** Oil Red O staining of 3T3-L1cells in both groups with using DMSO or AMPK inhibitor (compound C). **(B)** The mRNA expression of lipolysis and FAO genes in 3T3-L1 cells was performed under co-treatment of Chrysophanol with AMPK inhibitor (compound C). Chrysophanol, adipocytes were treated with a dose of 12.5 μM of Chrysophanol. All data are expressed as the mean ± SD (n=8).


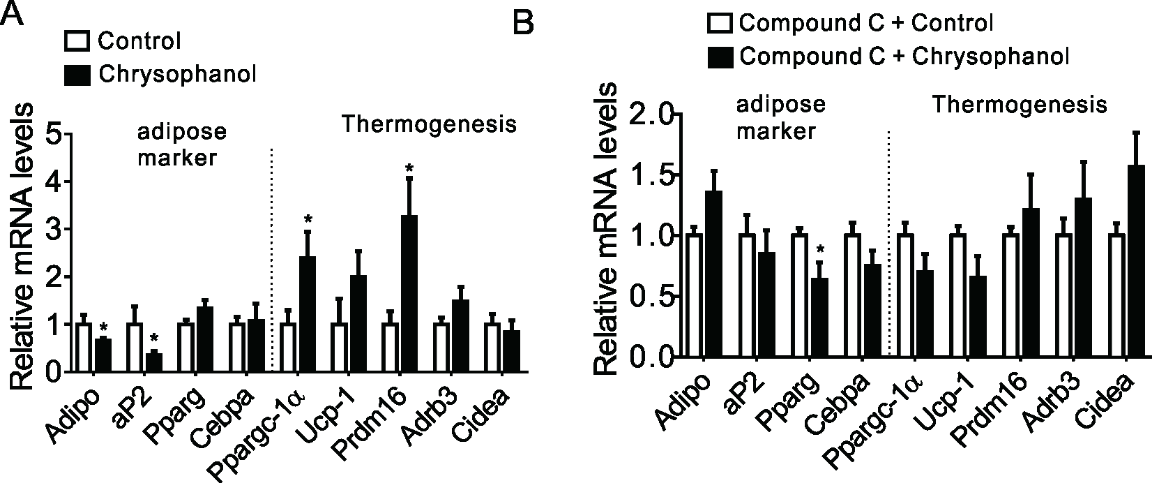


**Supplementary Figure 5: AMPK inhibitors attenuated the regulatory effect of Chrysophanol on adipose markers and thermogenic related genes expression.** **(A)** The mRNA expression of adipose markers and thermogenesis in 3T3-L1 cells was performed. **(B)** The mRNA expression of adipose markers and thermogenesis in 3T3-L1 cells was performed after using AMPK inhibitors (Compound C). Chrysophanol, adipocytes were treated with a dose of 12.5 μM of Chrysophanol. All data are expressed as the mean ± SD. ∗*p* < 0.05 compared with the control group (n=8).


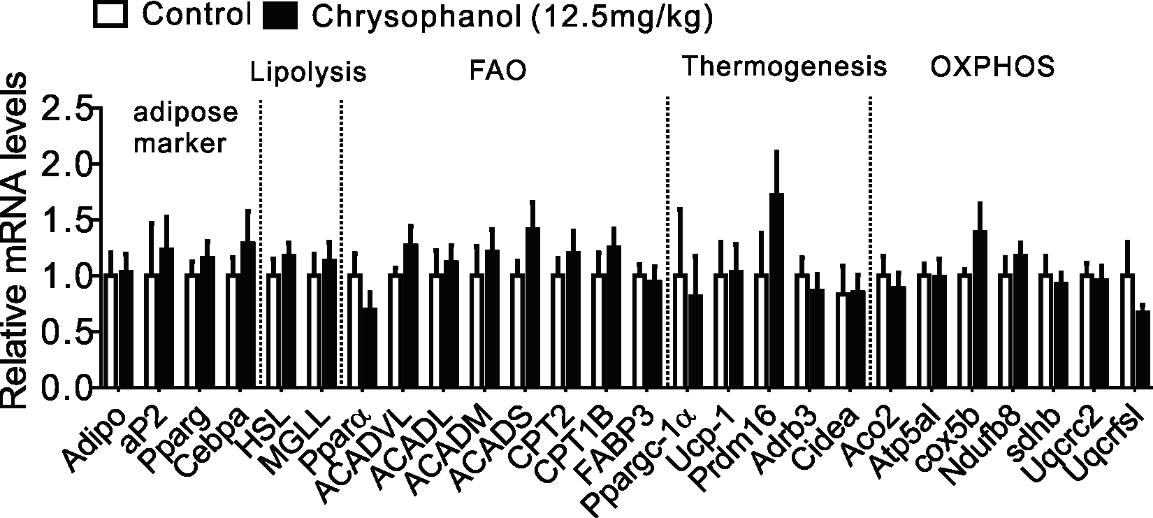


**Supplementary Figure 6: The effect of Chrysophanol in promoting thermogenesis was blunted in BAT of SRIT6 FKO mice.** The mRNA expression of adipose maker, lipolytic, FAO, thermogenesis, and OXPHOS in BAT of FKO mice was measured by real-time PCR. All data were expressed as the mean ± SD (n=8).
